# Comprehensive analysis of SSRs and database construction using all complete gene-coding sequences in major horticultural and representative plants

**DOI:** 10.1038/s41438-021-00562-7

**Published:** 2021-06-01

**Authors:** Xiaoming Song, Qihang Yang, Yun Bai, Ke Gong, Tong Wu, Tong Yu, Qiaoying Pei, Weike Duan, Zhinan Huang, Zhiyuan Wang, Zhuo Liu, Xi Kang, Wei Zhao, Xiao Ma

**Affiliations:** 1 School of Life Sciences/Library, North China University of Science and Technology, Tangshan, Hebei 063210 China; 2 School of Life Science and Technology and Center for Informational Biology, University of Electronic Science and Technology of China, 610054 Chengdu, China; 3 Food Science and Technology Department, University of Nebraska-Lincoln, Lincoln, NE 68588 USA; 4 College of Life Sciences and Food Engineering, Huaiyin Institute of Technology, 223003 Huai’an, China

**Keywords:** Genetic markers, Evolutionary biology, Genome

## Abstract

Simple sequence repeats (SSRs) are one of the most important genetic markers and widely exist in most species. Here, we identified 249,822 SSRs from 3,951,919 genes in 112 plants. Then, we conducted a comprehensive analysis of these SSRs and constructed a plant SSR database (PSSRD). Interestingly, more SSRs were found in lower plants than in higher plants, showing that lower plants needed to adapt to early extreme environments. Four specific enriched functional terms in the lower plant *Chlamydomonas reinhardtii* were detected when it was compared with seven other higher plants. In addition, Guanylate_cyc existed in more genes of lower plants than of higher plants. In our PSSRD, we constructed an interactive plotting function in the chart interface, and users can easily view the detailed information of SSRs. All SSR information, including sequences, primers, and annotations, can be downloaded from our database. Moreover, we developed Web SSR Finder and Batch SSR Finder tools, which can be easily used for identifying SSRs. Our database was developed using PHP, HTML, JavaScript, and MySQL, which are freely available at http://www.pssrd.info/. We conducted an analysis of the Myb gene families and flowering genes as two applications of the PSSRD. Further analysis indicated that whole-genome duplication and whole-genome triplication played a major role in the expansion of the Myb gene families. These SSR markers in our database will greatly facilitate comparative genomics and functional genomics studies in the future.

## Introduction

Since molecular marker technology was developed in the 1980s, an increasing number of molecular marker types have been identified, which has rapidly accelerated genetic improvements in species^1^. The development and comparative analysis of molecular markers could help us reveal genetic variation underlying various biological functional genes^2–4^. To date, researchers have found several molecular markers, such as restriction fragment length polymorphisms, random amplified polymorphism DNA, sequence tag sites, amplified fragment length polymorphism, diversity array technology markers, single-nucleotide polymorphisms, specific locus amplified fragments, and simple sequence repeats (SSRs)^1,5,6^.

These molecular markers play important roles in genetic map construction, quantitative trait locus detection, marker-assisted selection (MAS), and fine localization of important functional genes to fulfill various demands of breeders^7,8^. There have been many studies of molecular markers in model plants^1,9^. For example, several kinds of molecular markers were used to identify genes related to leaf senescence, leaf shape, chlorophyll, and embryogenesis in *Arabidopsis*^10–12^. Similarly, most genes determining disease resistance and major agronomic traits, such as grain quality, grain weight, and grain size, were also detected using molecular markers in rice^13–16^. In horticultural plants, molecular markers are also widely used for plant breeding in most species, including *Brassica rapa*, *Brassica oleracea*, *Solanum lycopersicum*, *Cucumis melo*, *Vitis vinifera*, *Fragaria ananassa*, and pear^17–22^. Furthermore, progress in molecular genetics, genomic selection, and genome editing has provided deep insights into the understanding of molecular markers and greatly complemented breeding strategies^1^.

SSR markers are present in almost all species, particularly in eukaryotes. These markers have many applications, such as constructing linkage maps, fine mapping of genes, and selective breeding through genomic selection^2,23–25^. SSRs have become extremely popular for phylogenetic analysis and have expanded our knowledge related to plant breeding^26–28^. The development of bioinformatics technology has enabled the development of SSR markers for many species^29–31^. Recently, there have been many reports on SSR development and application^32–38^. These studies have confirmed that SSRs are the classic, popular molecular markers used in plant science.

With an increasing number of plant genomes being released, it has become possible to construct a plant SSR database (PSSRD) using the SSRs identified from all genes in these plants. Compared with those in existing databases, all the species in the database in this study have undergone complete genome sequencing. In addition, the PSSRD provides primer information and Pfam function annotation, which allows researchers to use these SSRs in a more convenient manner than those in other databases. More importantly, we not only provide more comprehensive and representative SSR information with the construction of this database but also conduct large-scale systematic and comparative analyses of SSRs in 112 plants.

## Results

### Overview of the main interface of the PSSRD

**Fig. 1:**
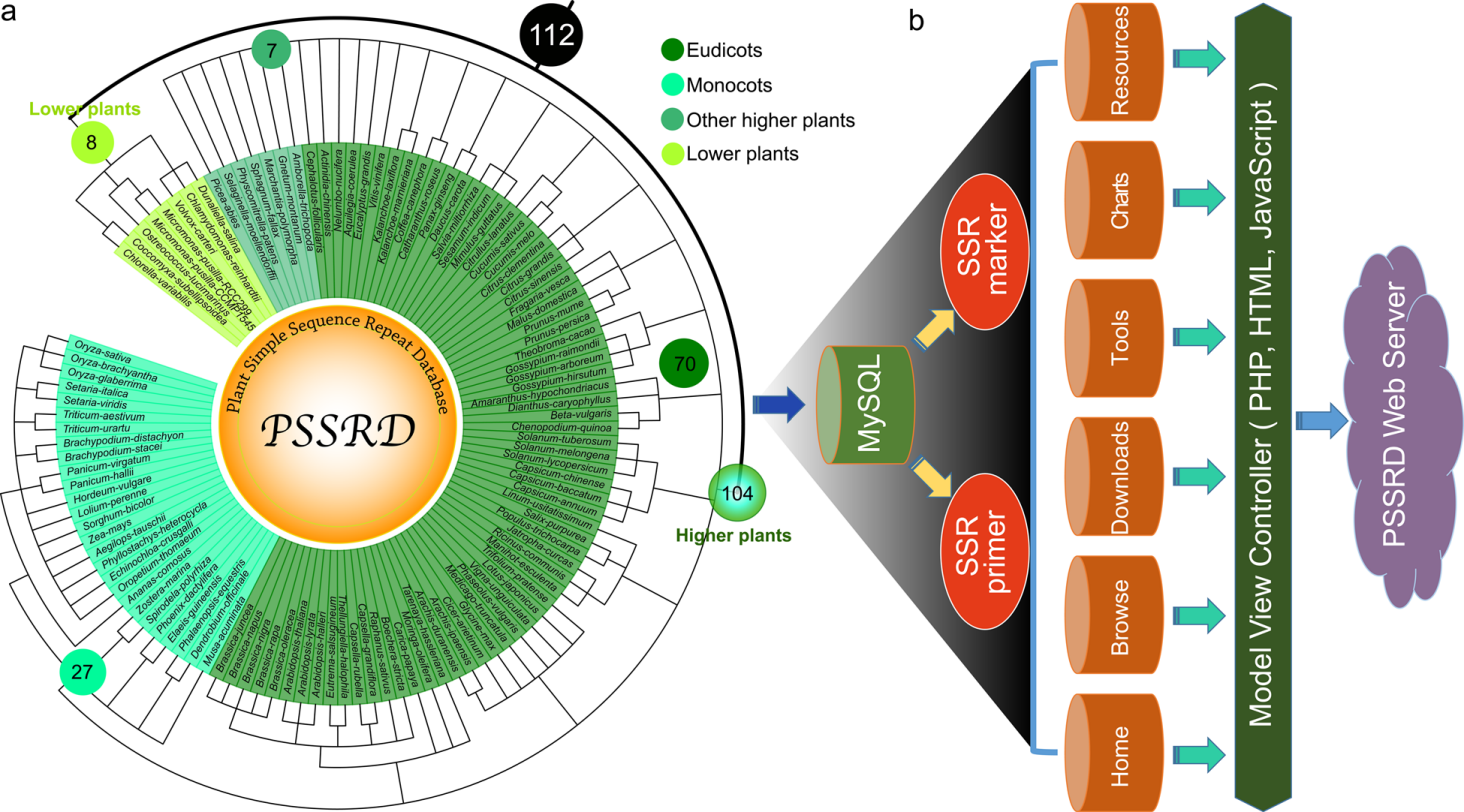
The architecture of the plant SSR database (PSSRD) and related species. **a** The phylogenetic relationship of 112 species used for constructing the PSSRD according to NCBI taxonomy^66^. **b** The PSSRD architecture mainly includes the home, browse, download, tool, chart, and resource interfaces

**Fig. 2:**
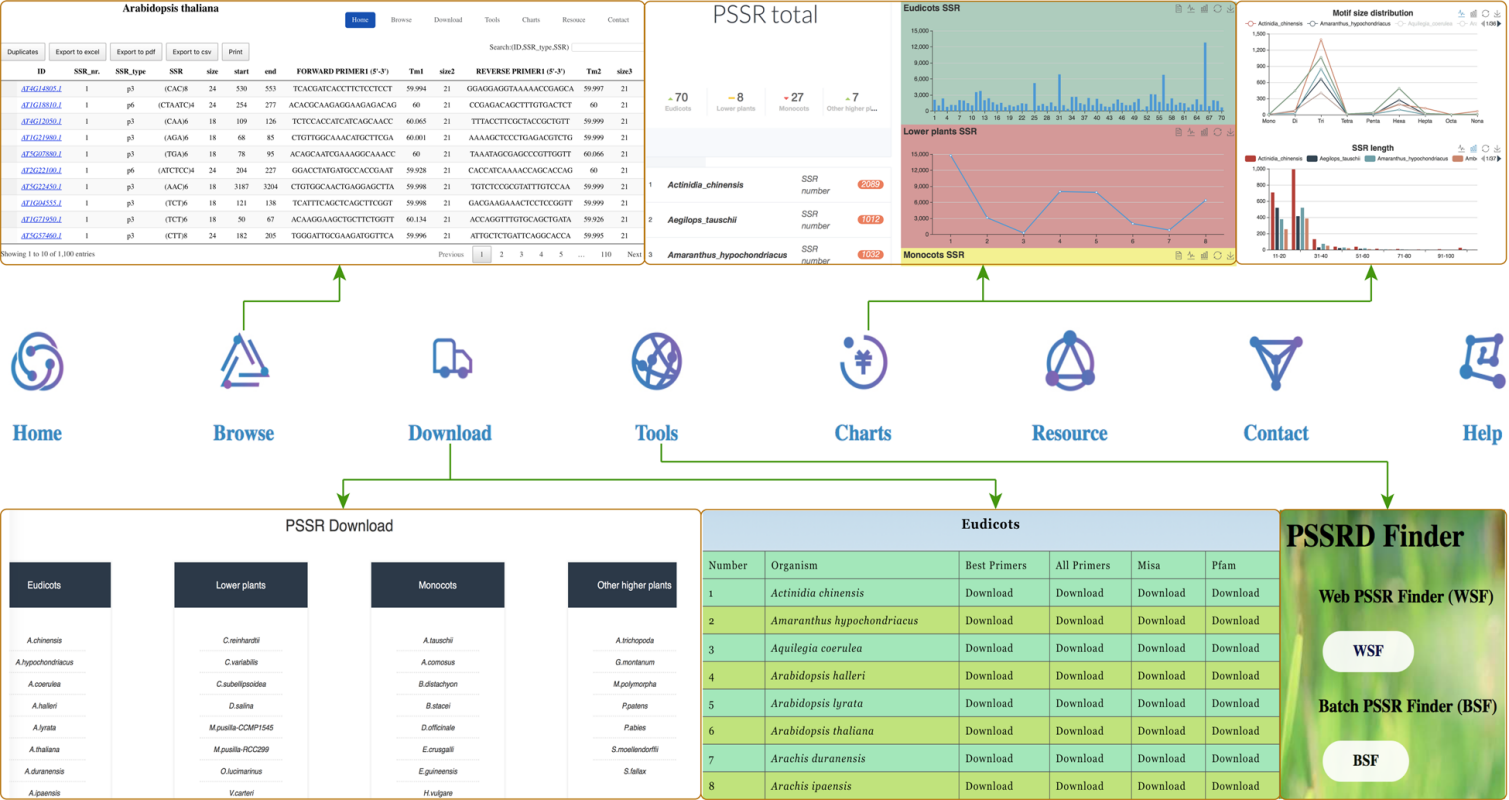
Overviews of the main interfaces and internal features of the plant SSR database (PSSRD). The overviews of the PSSRD mainly contain the home, browse, download, tool, chart, resource, contact, and help interfaces

We identified 249,822 SSRs from 3,951,919 gene sequences of 112 plant species. Specifically, 132,114, 64,980, 9478, and 43,250 SSRs were detected in 70 eudicots, 27 monocots, 7 other higher plants (1 basal angiosperm, 2 gymnosperms, 1 Lycopodiophyta, 2 Bryophyta, and 1 Marchantiophyta), and 8 lower plants, respectively (Fig. 1a and Table S1). Among these species, many are horticultural plants, such as vegetables (*B. rapa*, *Brassica oleracea*, *Capsicum annuum*, *Daucus carota*, and *S. lycopersicum*), fruits (*Citrus clementina*, *C. melo*, *Fragaria vesca*, *Prunus persica*, and *V. vinifera*), and flowers (*Prunus mume*, *Aquilegia coerulea*, and *Catharanthus roseus*). On average, primers were successfully designed for 98.82% of the SSRs for further study. Using these available datasets and related bioinformatics tools, we built a PSSRD, which helps users easily query, compare, and download SSR markers, primers, and functional annotations of several or all species simultaneously. All species used in this study were taxonomically classified to facilitate selection and use. The SSR information was stored in backend tables using MySQL (MySQL AB, Sweden) that can be accessed using the frontend web application of PSSRD (Fig. 1b). Here, we provide a detailed description of the interactive interfaces in this database, including the browse, chart, download, tool, resource, contact, and help interfaces (Fig. 2 and Fig. S1).

#### Browse

To make the database easy to use by researchers, we divided all species into different groups according to their taxa (Fig. 2). For each taxon, the species were further sorted by the first letter of their Latin names. We provided detailed information for each species, such as SSR information (type, sequences, size, start, and end), primer information (forward and reverse sequences, melting temperature (*T*_m_) value, and size), amplified production size, and related gene information (gene ID and links of Pfam annotation). Furthermore, we also integrated the search function at the browse interface, which allows the users to find related information according to gene ID, SSR type, and SSR sequences. Moreover, we provided a variety of export formats, including Excel, pdf, csv, duplicate, and print functions.

#### Chart interface

The chart interface provides several interactive plots to view the SSR data of all species (Fig. 2). First, the SSR number of each species is shown in the main interface, and the multiselect dropdown allows users to select the taxon for their needs. Furthermore, bar plots and line charts are used to show the SSR number of each species, which makes it easier and faster for users to compare SSRs between different species. Finally, all the information of these displayed SSRs can be downloaded at the lower-right corner of these pages as Excel files. These documents will allow researchers to conduct local batch SSR comparative analysis and perform relevant marker-assisted selective breeding experiments.

In addition, we provide further graphical representations of the SSR information for each species. Each species has six plots with pie charts, bar plots, and line charts, which show detailed information on SSRs, including SSR type, SSR length, product size, most frequent SSR, base number, and frequency of SSR distribution for each type. These diagrams could help users intuitively understand the SSR information of each species.

#### Download interface

The SSR information and statistics for each species can be downloaded from this interface (Fig. 2). Four files, including best primers, all primers, Pfam annotation, and position information of SSRs for each species, can be obtained from the download page of the PSSRD. The downloaded file is a tab-separated format, which can be browsed using Excel or other related text editors, such as EditPlus or Sublime text.

#### Tool interface

In addition to providing SSR information retrieval, graphical display, and download services for existing species, we developed two tools, the Web SSR Finder (WSF) and Batch SSR Finder (BSF) programs (Fig. 2). These two tools can assist researchers in conducting SSR identification and analysis for a new species.

For the WSF, users can upload nucleic acid sequences in the FASTA format and then set the minimum number of repetitions for various types of SSRs. Finally, the start button can be clicked, and after a moment, the relevant SSR identification results are obtained.

The BSF program can batch-detect SSRs in multiple species on the local server. Although the previous MISA program could identify SSRs, it only detected the SSR of one species at a time. Therefore, we have modified and updated the MISA program and named the new program BSF. In addition to some basic SSR identification files, we also provide comparative analysis files of SSRs between different species. With the completion of additional genome sequencing, a batch-comparison study needs to be conducted on the SSR information of a large number of species. Therefore, the updated BSF program is more convenient for users to carry out batch SSR identification and multispecies comparative studies. Anyone engaged in scientific research can download and freely use or further edit this program according to their own analysis needs.

#### Resource, help, and contact interfaces

For the resource interface, we collected most of the SSR research-related databases and provide relevant links for users to easily query and compare studies (Fig. 2). For the help interface, we provide the researcher with a detailed PSSRD user manual. In addition, we provide contact information to help users contact us conveniently and quickly.

### Comprehensive comparative analysis of the SSRs in 112 species

#### Trinucleotide SSRs were dominant according to the frequency distribution analysis

**Fig. 3:**
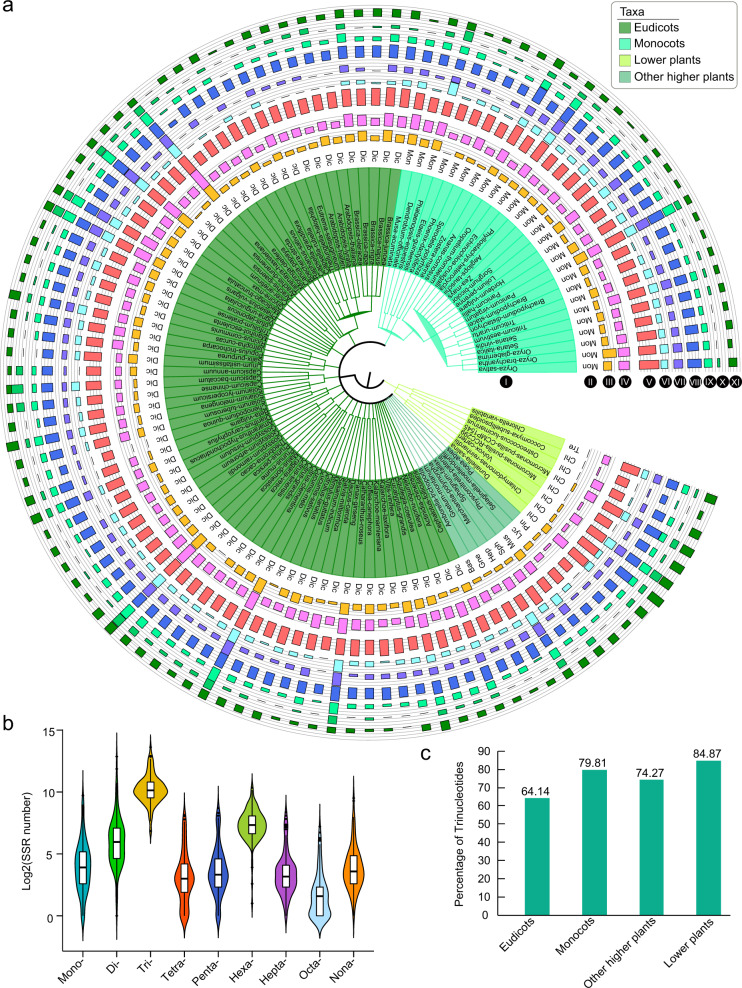
The number of each SSR type and SSR number were log_2_ transformed. **a** The number of each SSR type in 112 plants. I, plant Latin name and taxa; II, subtaxa; III–XI, the histogram of the numbers for the SSR types from mono- to nonanucleotides, respectively. **b** Boxplot of the number of each SSR type in 112 plants. **c** The percentage of the trinucleotide SSR type in eudicots, monocots, other higher plants, and lower plants

In our study, all the SSRs were divided into nine types from mono- to nonanucleotides (Fig. 3a and Table S1). We found that trinucleotides were the most common SSR type in all four groups, and the average percentages of the SSR numbers were 64.14%, 79.81%, 74.27%, and 84.87% for eudicots, monocots, other higher plants, and lower plants, respectively (Fig. 3c). Nevertheless, we found that the number of trinucleotide SSRs varied considerably among different species, ranging from 114 (eudicot plant: *Chenopodium quinoa*) to 12,663 (lower plant: *C. reinhardtii*). The average number of trinucleotide SSRs was 1610 in 112 plants, followed by dinucleotide SSRs (229) and hexanucleotide SSRs (219) (Fig. 3 and Table S1). This result might have occurred because the trinucleotides in the gene-coding regions did not lead to the transcoding of genes. This theory could be further verified by considering hexanucleotides, the percentage of which was also greater than that of the other SSR types in the four groups (Fig. 3b).

#### Correlation analysis of the factors related to different SSR characteristics

To explore the relationship between the factors related to different SSR characteristics, we conducted a correlation analysis for these factors. Here, we investigated several factors related to SSR characteristics, including SSR number, SSR density (SSR number per Mb), number of genes containing SSRs, and percentage of genes containing SSRs. In addition, the factors total gene number and total length of gene sequences were also used for the comparative analysis in all examined plants.

**Fig. 4:**
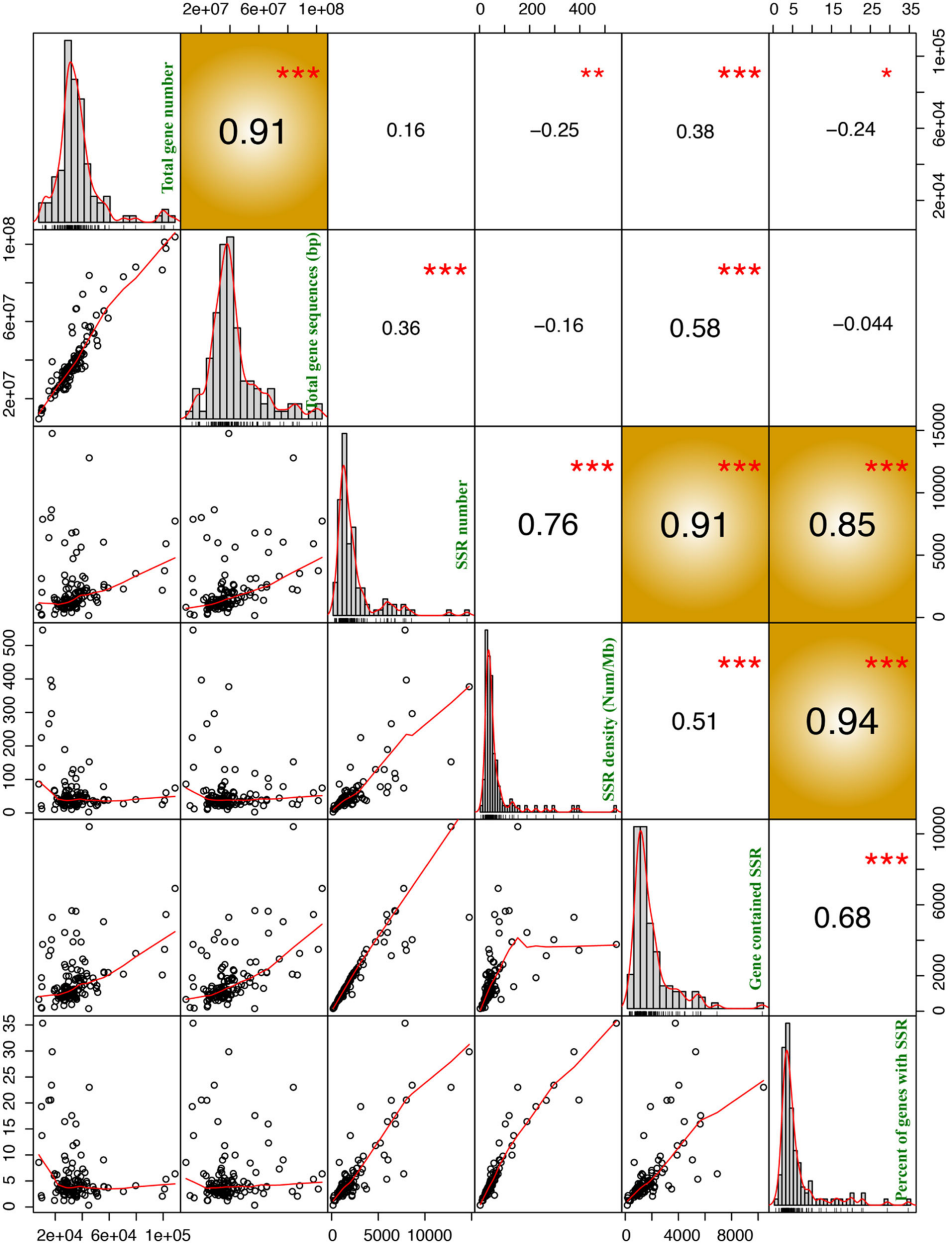
Correlation analysis of different SSR characteristics, including total gene number, total gene sequences, SSR number, SSR density, number of genes containing SSRs, and percentage of genes containing SSRs. The lower-left corner represents the correlation analysis scatter diagram for different SSR characteristics. The plots in the middle are a bar chart for each SSR feature. The upper-right corner represents the correlation values between different SSR features. The 1, 2, and 3 red asterisks represent *P* < 0.05, *P* < 0.01, and *P* < 0.0001, respectively. The yellow background represents the SSR characteristics with significant differences (correlation coefficients > 0.80 and *P* value < 0.0001)

A significant correlation was detected between the percentage of genes containing SSRs and the SSR number or SSR density in plants (correlation coefficients > 0.80 and *P* value < 0.01) (Fig. 4). However, there was no significant correlation between SSR number and total gene number or the total length of gene sequences.

#### Comparative analysis indicated that more SSRs were present in lower plants than in higher plants

**Fig. 5:**
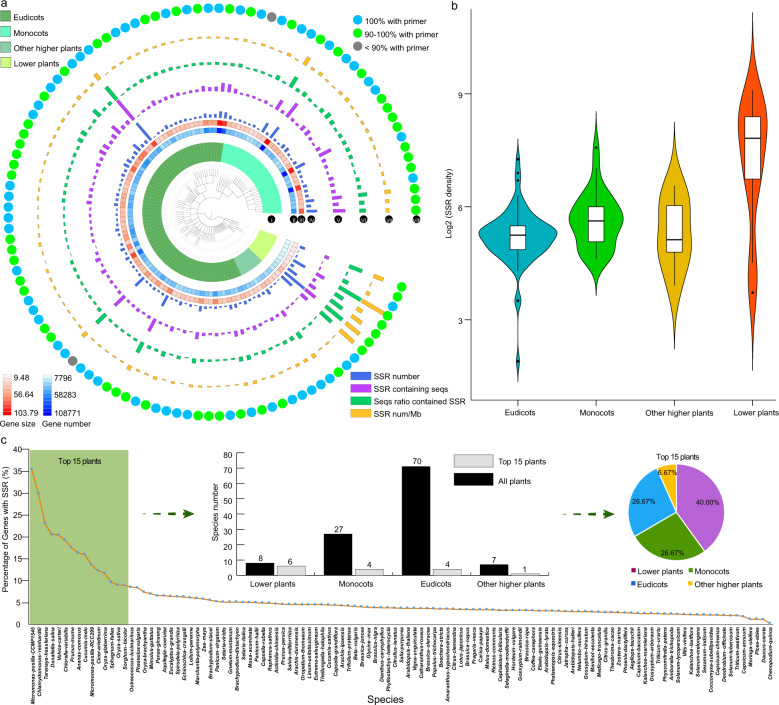
Comparative analysis of different SSR characteristics. **a** Statistical analysis results of the different SSR characteristics in 112 species. I, plant classification; II, heatmap of total gene number; III, heatmap of total gene sequences; IV, a bar chart of SSR number; V, number of genes containing SSRs; VI, percentage of genes containing SSRs; VII, SSR density; and VIII, the status of SSRs with primer. **b** The boxplot of SSR density in four groups. **c** The statistical analysis results for the percentage of genes containing SSRs in all species and the top 15 species

Our analyses showed that among the plants, the different lower plants had the largest SSR variations, including variations in SSR number, SSR density, number of genes containing SSRs, and percentage of genes containing SSRs (Fig. 5a, b and Fig. S2). The average SSR density in lower plants was the largest (256.90), followed by that in monocots (55.92), other higher plants (46.34), and eudicots (40.54) (Table S1).

To obtain detailed information about the SSRs in each species, we carried out a further analysis. Overall, more SSRs were detected in lower plants than in higher plants (Fig. 5). Among the top 15 species with a high percentage of genes containing SSRs, six (40.00%) species belonged to lower plants (Fig. 5c). Two species with the highest percentage of SSR genes were lower plants, *Micromonas pusilla* CCMP1545 and *C. reinhardtii* (Fig. 5c and Table S1). In *M. pusilla* CCMP1545, 3768 genes contained SSRs, accounting for 35.35% of the total number of genes. This result might have been due to the special role played by SSRs in lower plants and provides a new perspective for the study of SSR function.

However, there were some exceptions in higher plants, which also had high SSR ratios. For example, in eudicots, the highest percentage of genes containing SSRs (23.02%) was found in spider flowers (*Tarenaya hassleriana*), with 12,799 SSRs, followed by *P. mume* (17.58%) and *C. melo* (15.96%) (Fig. 5c and Table S1). In monocots, the highest percentage of SSR genes (16.42%) was found in pineapple (*Ananas comosus*) with 5991 SSRs, followed by *Oryza glaberrima* (11.80%) and *Oryza sativa* (9.05%) (Fig. 5c and Table S1).

#### Functional enrichment analysis of genes containing SSRs in 112 species

To further explore the function of SSRs, we conducted functional annotation using the Pfam database. A total of 69.75% of the annotated genes contained SSRs in monocots, followed by those in eudicots (69.25%), other higher plants (65.29), and lower plants (60.27%) (Table S2). We further performed functional enrichment analysis of these SSR-related genes in 112 plants, and 155 terms were enriched with a *q* value < 0.05 and fold change ≥2 (Table S3). Our enrichment analysis required that the annotation ratio of the term for SSR genes was twice as high as that of the whole-genome genes. The most enriched term was AP2, followed by Myb_DNA-bind 4, Myb_DNA binding, and TCP family genes. Interestingly, we found that the most significantly expanded terms belonged to the transcription factors associated with the regulation of abiotic stress, such as Myb, TCP, AP2, WRKY, and various zinc-finger (zf-CxHx) proteins (Table S3). This result indicated that SSRs might play a very important role in the regulation of plant stress.

**Fig. 6:**
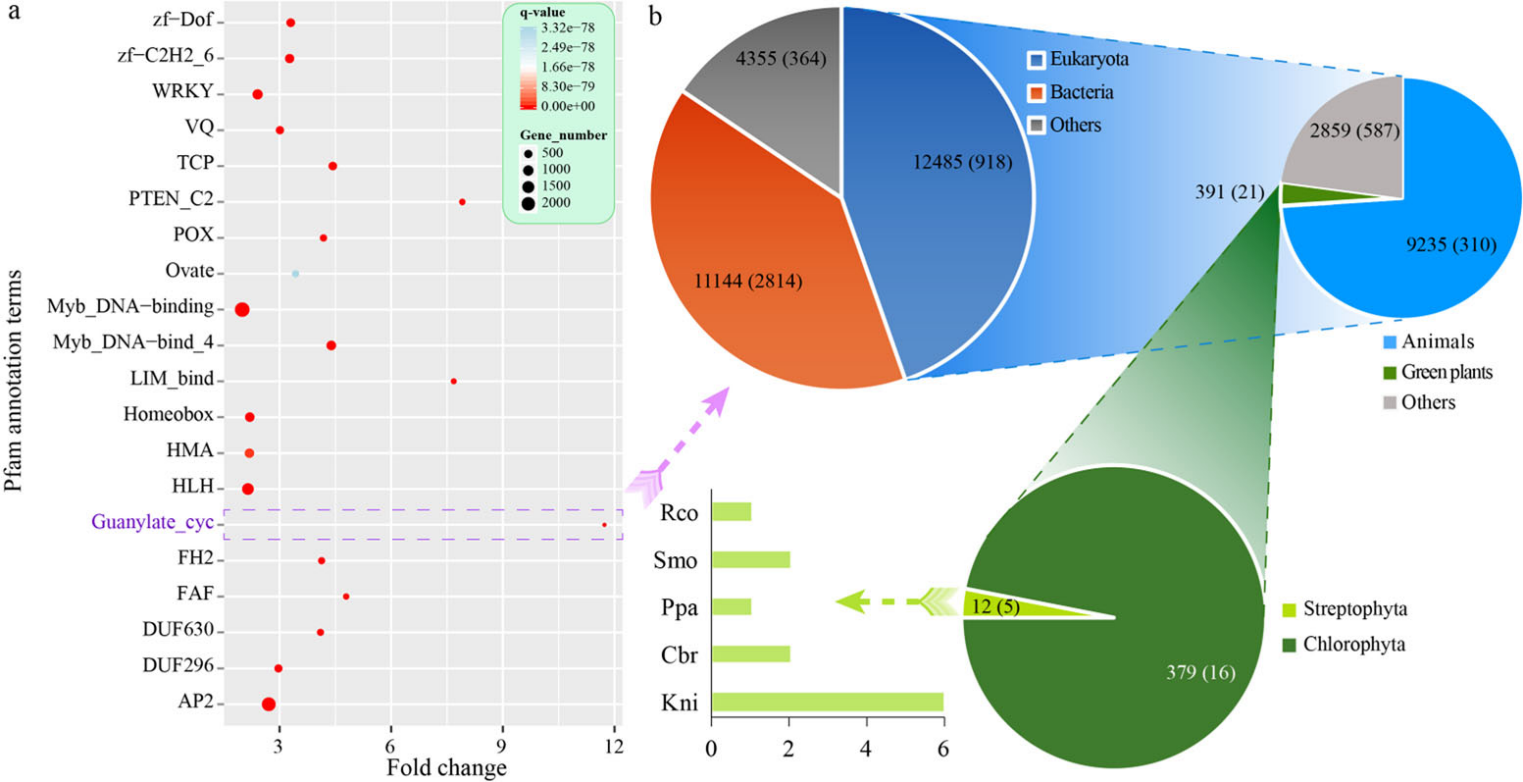
Functional enrichment analysis of SSR-related genes in 112 species. **a** The top 20 enriched terms based on Pfam annotation. The size of the dot indicates the number of enriched genes in the related pathway, and the color of the dot corresponds to different *q* value ranges. **b** The gene number with the Guanylate_cyc (PF00211) domain in different kinds of species according to the Pfam database. The numbers within parentheses denote the number of species for each taxon. Rco *Ricinus communis*, Smo *Selaginella moellendorffii*, Ppa *Physcomitrella patens*, Cbr *Chara braunii*, Kni *Klebsormidium nitens*

Furthermore, we selected the 20 most significantly enriched terms for graphic presentation, and all had *q* values < 3.32e − 78 (Fig. 6a). Among the 20 top enriched terms, the largest fold change was over 11.73 for Guanylate_cyc, followed by that for PTEN_C2 (7.91) and LIM_bind (7.68). This result indicates that these enriched proteins might play critical roles through SSRs in plants.

Further analysis showed that Guanylate_cyc (PF00211) was found in 27,984 sequences from 4096 species according to the Pfam database. Among these sequences, 12,485 sequences from 918 species belonged to Eukaryota, while most of the other sequences belonged to bacteria (Fig. 6b and Fig. S3). In Eukaryota, most sequences (9235) were from 310 species of Metazoa, while only 391 sequences belonged to 21 species of green plants (Viridiplantae). In Viridiplantae, 12 sequences were from five Streptophyta species, and 379 sequences were from 16 Chlorophyta species (Fig. 6b). Therefore, more genes containing the Guanylate_cyc domain were found in lower plants than in higher plants.

Among the five species from Streptophyta, two species belonged to Charophyta (*Klebsormidium nitens* and *Chara braunii*), which contained six and two genes with the Guanylate_cyc domain, respectively (Fig. 6b). The other three species were from land plants, including one Bryophyta (*Physcomitrella patens*), one Lycophyte (*Selaginella moellendorffii*), and one angiosperm (*Ricinus communis*). All identified SSRs located in these genes with the Guanylate_cyc domain could be used as markers for functional studies in the future.

#### Functional enrichment analysis of genes containing SSRs in eight representative species

**Fig. 7:**
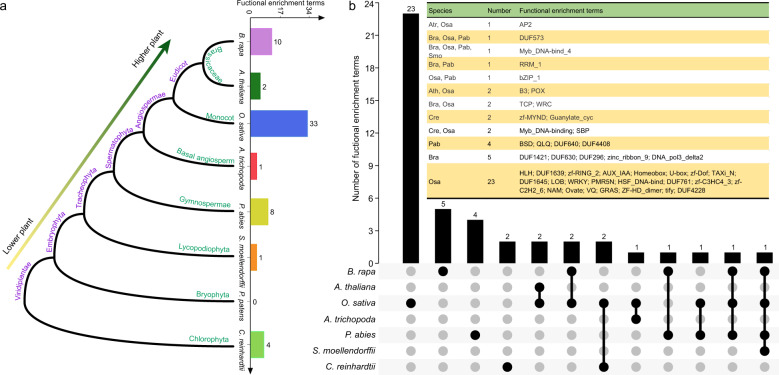
Functional enrichment analysis of genes containing SSRs compared with whole-genome genes in eight representative species. **a** The phylogenetic tree and the number of enriched functional terms (*q* value < 0.05, fold change ≥ 2) for eight species. **b** The Venn diagram shows the common and specific enriched functional terms based on the Pfam database in eight representative species. The abbreviations are as follows: Cre *C. reinhardtii*, Bra *B. rapa*, Ath *A. thaliana*, Osa *O*. *sativa*, Atr *A. trichopoda*, Pab *P. abies*, Smo *S. moellendorffii*, Ppa *P. patens*

We further explored the function of genes containing SSRs in eight representative lower plants (Chlorophyta: *C. reinhardtii*) and higher plants, including the horticultural plant *B. rapa*, eudicot model plant *Arabidopsis thaliana*, monocot model plant *O*. *sativa*, basal angiosperm *Amborella trichopoda*, gymnosperm *Picea abies*, Lycopodiophyta *S. moellendorffii*, and Bryophyta *P. patens* (Fig. 7a).

Based on the Pfam functional annotation, we performed an enrichment analysis of genes containing SSRs and compared them with whole-genome genes in eight representative plants (*q* value < 0.05, fold change ≥ 2). The most significantly enriched functional terms were detected in *O*. *sativa* (33), followed by in *B. rapa* (10), *P. abies* (8), *C. reinhardtii* (4), *A. thaliana* (2), *A. trichopoda* (1), and *S. moellendorffii* (1) (Fig. 7a and Table S4). However, no enriched functional terms were found in *P. patens*.

Further Venn diagram analysis showed 23, 5, 4, and 2 enriched functional terms specific to *O*. *sativa*, *B. rapa*, *P. abies*, and *C. reinhardtii*, respectively (Fig. 7b). Two specific functional terms for the lower plant *C. reinhardtii* were zf-MYND and Guanylate_cyc (Fig. 7b). This result was also consistent with the above analysis of the Guanylate_cyc domain; that is, this domain mainly existed in lower plants. Interestingly, we found that Myb_DNA-bind_4 was detected in most plants as an enriched functional term, including *B. rapa*, *O*. *sativa*, *P. abies*, and *S. moellendorffii*. In addition, Myb_DNA binding was enriched in *O*. *sativa* and *C. reinhardtii*. This phenomenon indicated that Myb-related genes might play important roles mediated by SSRs in plants.

### PSSRD application 1: Myb-related gene families

#### Phylogenetic and comparative analysis of Myb-related gene families

Since the above analysis showed that Myb family genes were significantly enriched in SSR-related genes, we further conducted phylogenetic and comparative analysis of several Myb gene families.

**Fig. 8:**
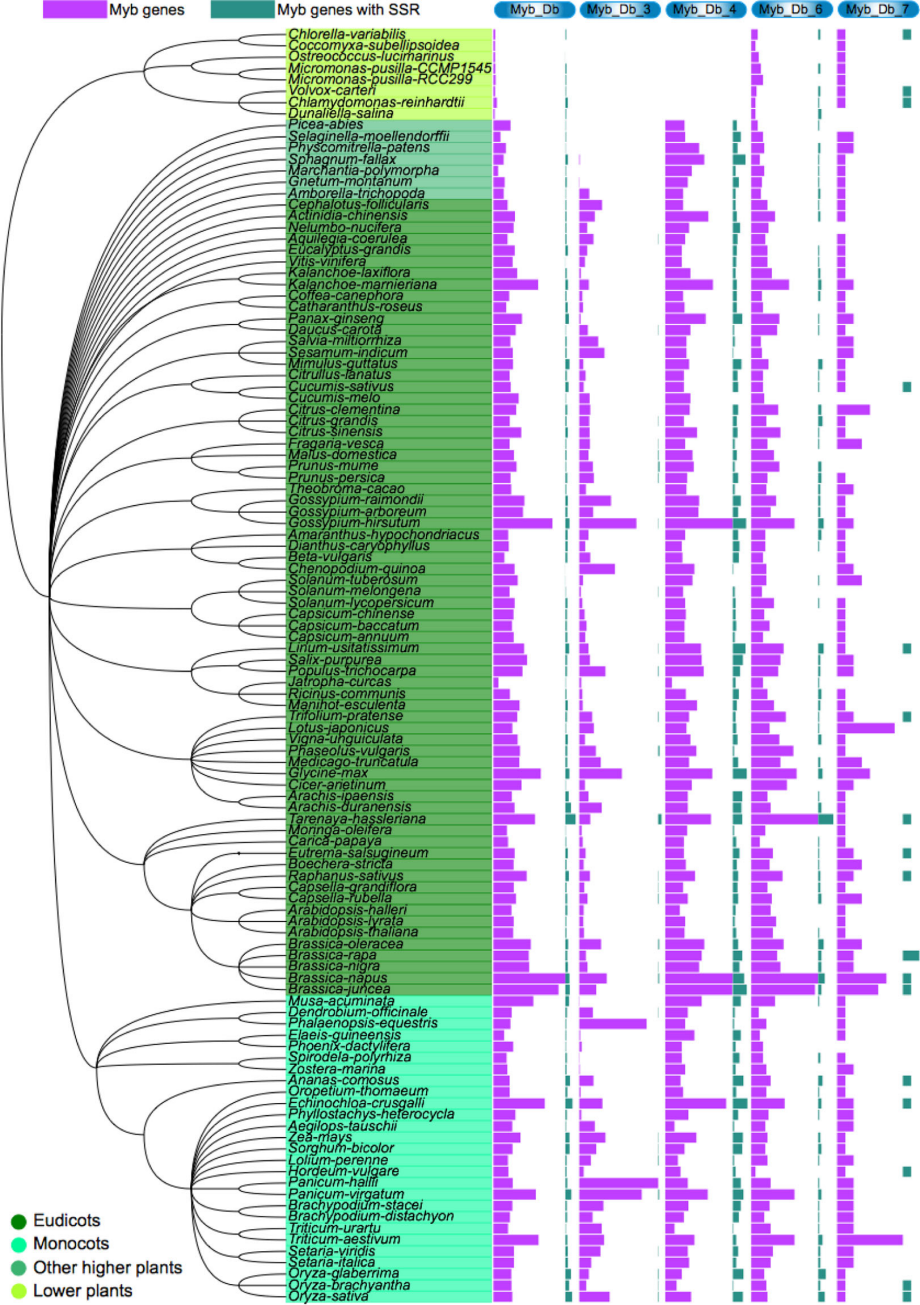
Plot of gene number for five Myb-related gene families (Myb_DNA-binding, Myb_DNA-bind_3, Myb_DNA-bind_4, Myb_DNA-bind_6, and Myb_DNA-bind_7) in 112 species. The bar chart with purple bars indicates the Myb-related genes and the green indicates the Myb-related genes that contained SSRs

Based on the Pfam annotation of whole-genome genes from 112 species, we identified 38,982 Myb-related genes from five gene families, including 28,741 Myb_DNA binding, 3979 Myb_DNA-bind_3, 4,034 Myb_DNA-bind_4, 2,056 Myb_DNA-bind_6, and 172 Myb_DNA-bind_7 family genes (Fig. 8 and Tables S5–9). Our analysis showed that Myb_DNA binding and Myb_DNA-bind_6 family genes were present in 112 plants, while Myb_DNA-bind_3, Myb_DNA-bind_4, and Myb_DNA-bind_7 family genes were only detected in 100, 104, and 103 plants, respectively. In particular, there were no Myb_DNA-bind_3 or Myb_DNA-bind_4 family genes in the eight examined lower plants (Fig. 8 and Tables S5–9). Compared with the other four families, the Myb_DNA-bind_4 gene family had the highest proportion of SSRs in most plants, with an average ratio of over 20.73%.

**Fig. 9:**
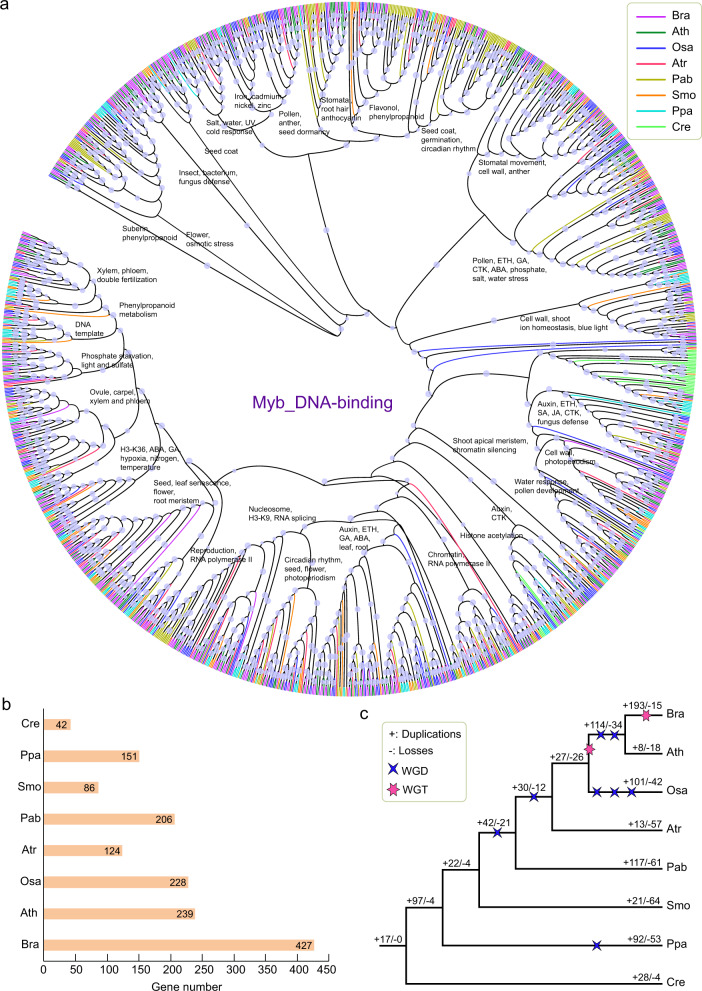
Phylogenetic and gene duplication or loss analysis of the Myb_DNA-binding gene family in eight representative species. **a** Maximum-likelihood (ML) trees were generated based on the amino acid sequences of the Myb_DNA-binding gene family. The phylogenetic tree was constructed using FastTree software with 1000 bootstrap repeats in eight species. Bootstrap values >40% are shown with circles on each branch. **b** The gene number of the Myb_DNA-binding gene family in each species. **c** Gene duplication and loss analyses of the Myb_DNA-binding gene family using the Notung software in eight species. Differential gene duplications and losses are indicated by numbers with a + and − on each branch. Whole-genome duplication (WGD) and whole-genome triplication (WGT) events are indicated with a quadrilateral and hexagon, respectively

To explore the evolution and function of Myb gene families, we constructed a phylogenetic tree using Myb-related genes from five families in eight representative species, including *B. rapa*, *A. thaliana*, *O*. *sativa*, *A. trichopoda*, *P. abies*, *S. moellendorffii*, *P. patens*, and *C. reinhardtii* (Fig. 9 and Fig. S4–7). According to the topology of the phylogenetic tree, the genes of each Myb-related gene family were classified into different groups. We marked the main functions of most groups according to the Myb family gene functions in *Arabidopsis*. This result provided a good reference for studying other genes with unknown functions in the same group. Interestingly, we found that most Myb_DNA-binding family genes of the lower plant *C. reinhardtii* were clustered on the same branch in the evolutionary tree, while the genes of the other seven species were scattered on different branches (Fig. 9a). This result indicated that the genes of this gene family have experienced changes in the base sequences or gene structure. Thus, Myb_DNA-binding family genes might have evolved to have a greater variety of functions in higher plants than in lower plants, which might have allowed higher plants to become better adapted to terrestrial environments. In addition, we performed a comprehensive analysis of four other Myb-related gene families (Figs. S4–7).

#### Gene duplication and loss inference of Myb-related gene families

We analyzed the duplication and loss of Myb-related gene families in these eight plants using the Notung software through reconciliation between species and gene phylogenetic trees.

Among the eight species, the most genes were identified in *B. rapa* for all five Myb gene families (Fig. 9, Figs. S4–7, Table S10). In *B. rapa*, the number of Myb_DNA-binding family gene duplications was higher than the number of gene losses (193 vs. 15), whereas in *Arabidopsis*, the number of gene duplications was lower than the number of gene losses (Fig. 9c). *Brassica rapa* underwent an additional whole-genome triplication (WGT) event since its divergence from *Arabidopsis* according to a previous report^39^. Therefore, we inferred that WGT events might play important roles in the expansion of the Myb_DNA-binding gene family in *B. rapa*.

Similarly, there were more gene duplications than gene losses in *O. sativa* and *P. patens*, and these duplications occurred in one or several whole-genome duplication (WGD) events. For the other four Myb gene families, we found that they had similar trends in gene duplications and losses as those of the Myb_DNA-binding gene family (Figs. S4–7). Therefore, we believe that WGD or WGT plays a major role in the expansion of Myb gene families. This finding provides new insights and guidance into SSRs and other gene family analyses using datasets from our PSSRD.

### PSSRD application 2: flowering-time gene analysis

SSRs are often located in some important functional genes related to plant development and various abiotic stress responses^2,40,41^. Here, we took flowering-time genes as an example to show the application of SSRs stored in our PSSRD. In plants, flowering is critically important for successful sexual reproduction and fruit and seed development^42,43^. A diverse range of environmental and endogenous signals regulate flowering^44,45^. Previous reports have indicated that many genes are involved in regulating plant flowering, and they could be assigned to several regulatory pathways, including photoperiod, vernalization, gibberellin, ambient temperature, autonomous, and aging pathways^43,46^.

**Fig. 10:**
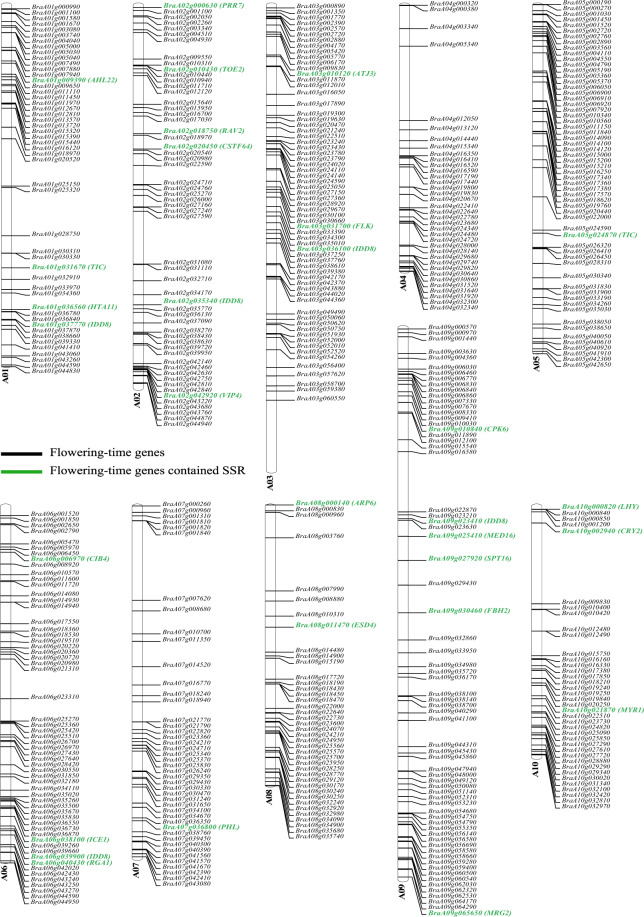
The distribution of flowering-time genes on ten chromosomes in *B. rapa*. The green indicates that the flowering-time genes contained SSR markers

Most flowering-time genes have been reported and functionally characterized in *Arabidopsis* and *Brassica* species^42,43,46–48^. In *Arabidopsis*, 306 flowering-time genes have been identified, including 295 coding and 11 noncoding genes according to previous reports^47,48^. Based on these coding genes, we identified 514 homologous flowering-time genes in the horticultural plant *B. rapa* when compared with those in *Arabidopsis* by the Blastp program (Fig. 10 and Table S11). Further analysis showed that 30 genes contained SSRs, accounting for 5.84% of all 514 flowering-time genes in *B. rapa* (Fig. 10). For example, the flowering locus KH domain (*FLK*, *BraA03 g031700*), phytochrome-dependent late flowering (*PHL*, *BraA07 g036800*), and cryptochrome 2 (*CRY2*, *BraA10 g002940*) genes contained SSRs in *B. rapa*. These SSRs will be useful for MAS breeding for flowering in *Brassica* in the future. Similarly, users could also search for SSRs in other functional genes of 112 species from the PSSRD. Therefore, our database can provide researchers with plentiful SSR resources.

The distribution of flowering-time genes on ten chromosomes in *B. rapa*. The green indicates that the flowering-time genes contained SSR markers.

## Discussion

In this study, we comprehensively identified SSRs from all the gene-coding sequences (CDSs) of 112 plants and further performed functional enrichment analysis for SSR-related genes. Among the top 20 significant functional enrichment terms, the Guanylate_cyc term had the largest fold change for SSR-related genes relative to the whole-genome level. Interestingly, further investigation showed that the Guanylate_cyc domain existed in lower plants and other nonplant species, while it was rarely found in higher plants. Based on previous reports, guanylate cyclases catalyze guanosine triphosphate to cyclic guanosine monophosphate (cGMP). As an intracellular messenger, cGMP activates kinases and regulates ion channels^49,50^. Guanylate cyclases are part of the G-protein signaling cascade, which is inhibited by high intracellular calcium levels but activated by low calcium levels^51,52^. Therefore, the genes with the Guanylate_cyc domain might play critical roles in lower plants, animals, and bacteria. This finding provides a new perspective for the functional study of SSR-related genes.

Our findings showed that the most significantly expanded functional terms were transcription factor families related to the regulation of abiotic stresses, such as Myb, AP2, and WRKY. Most of these gene families played important roles in stress resistance in plants according to previous reports^53–57^. This result indicated that SSRs might play critical roles in regulating plant stresses. Further comparative analysis of eight representative plants showed that several specific and common enriched functional terms were detected. Among all functional enriched genes, Myb-related gene families existed in most plants. The Myb gene family has a wide range of effects on plant growth, development, and stress resistance, such as anther development, axillary meristem formation, cell-wall thickening, and sperm cell formation^58–60^. The Myb gene family is also involved in several biosynthesis pathways, such as anthocyanin and flavonol synthesis, and hormone responses^59,61,62^. Our further analysis indicated that WGD and WGT played a major role in the expansion of the Myb gene families. This finding provided new insights and guidance into SSRs and other gene families.

Currently, an increasing number of genomes have been sequenced, and it is possible to develop a large number of SSR markers at the whole-genome level in different species from each main kingdom. To date, several databases have been constructed to collect SSRs from one or more species, such as the Plant Microsatellite Database, FishMicroSat, and Microsatellite Database^63–65^. However, most existing SSR databases were constructed several years ago and have not been updated with novel sequence information, or they cannot be accessed. Therefore, we constructed a PSSRD in this study, and it will be updated with new SSR datasets and information promptly in the future. With the increasing number of genome sequences released, we will continuously collect novel genomic datasets and identify SSRs and store them in our PSSRD for users. We also encourage users to submit their new SSR datasets to us to further enrich and refine the database. Moreover, we welcome all users to send us feedback for further improvement of our database. We believe that the PSSRD will be a useful and user-friendly database for researchers.

## Conclusion

In conclusion, we constructed a PSSRD for widely collected SSR sequences from 112 plants. Interestingly, we found that more SSRs were detected in the lower plants than in the higher plants. Moreover, a comprehensive comparative analysis of SSRs was conducted to reveal their basic characteristics and functional enrichment in different plants. This PSSRD can be used for comparative genomic analysis and molecular MAS studies of plants in the future.

## Materials and methods

### Sequence collection

The CDSs and protein sequences of each plant in Fasta format were downloaded from the ensemble database (http://useast.ensembl.org/index.html). The alternative splice sequences within the species were removed by custom Perl script to ensure no redundancy of the datasets. We have provided detailed information on the 112 plants used in this study, such as the classification, genome information, and related references in Table S12. Based on the relationship of these species in the NCBI taxonomy, the phylogenetic trees were further edited and shown using the iTOL program^66,67^.

### Identification and characterization of SSRs

The SSRs of the gene sequences in the selected species were identified using a batch SSR search program, which was written according to the Microsatellite identification tool (MISA)^68^. The parameters were set as follows: monomers (×16), 2-mers (×8), 3-mers (×6), 4-mers (×5), 5-mers (×4), 6-mers (×4), 7-mers (×3), 8-mers (×3), and 9-mers (×3)^69^. This program allowed the identification and localization of perfect and compound microsatellites. When the sequence length between two SSRs was <100 bp, we defined them as a compound SSR according to previous reports and the default parameters of the MISA software^70,71^.

### Primer design for SSR markers

The primers were designed for the identified SSRs using the Primer3 program^72^. The main parameters were set as follows according to a previous report^2^: (i) the optimum primer length was 20 nucleotides, and the range was from 18 to 27 bases. (ii) The optimum temperature of the *T*_m_ was 60 °C, and the range was from 55 to 65 °C. (c) The optimum size of the target PCR products was 150 bp, and the range was from 100 to 280 bp. All other parameters were set to the default values according to the Primer3 program.

### SSR statistics and correlation analysis of different factors

Violin plots with boxplots of SSR number, SSR density, and the percentage of genes containing SSRs were drawn using the ggviolin function in the ggpubr package of the R program (https://cran.r-project.org/web/packages/ggpubr/index.html). Correlation coefficients and significance tests were performed using the Hmisc and Performance Analytics packages of the R program (https://www.r-project.org/). The definition of significant correlation was an absolute value of correlation coefficients > 0.80 and a *P* value < 0.01.

### Functional annotation and enrichment analysis

The functional annotation of the genes containing SSRs and all other genes was conducted using the localized Pfam database (http://pfam.sanger.ac.uk)^73^. The Venn diagram was drawn by TBtools^74^. The functional enrichment analysis of the SSR-related genes compared with the whole-genome genes was conducted using the SciPy package of Python^75^. Then, R was used to perform Benjamini and Hochberg correction on the *P* value of significance test, and the parameters for significant functional enrichment terms were defined as *q* value < 0.05 and fold change ≥ 2^76,77^.

### Identification and analysis of important functional gene families

Pfam was used to perform a domain search on the amino acid sequences of each species. The genes containing the domains of “Myb_DNA binding” (PF00249), “Myb_DNA-bind_3” (PF12776), “Myb_DNA-bind_4” (PF13837), “Myb_DNA-bind_5” (PF13873), “Myb_DNA-bind_6” (PF13921), and “Myb_DNA-bind_7” (PF15963) were extracted by self-programmed Perl with an *e* value <1e − 4. In addition, the Simple Modular Architecture Research Tool and Conserved Domains Database were used to conduct domain validation on these genes to ensure accuracy^78,79^. *Arabidopsis* flowering genes were collected from FLOR-ID and previous reports^47,48^. The homologous flowering genes in *B. rapa* were identified by a comparison with those in *Arabidopsis* by the Blastp program (*e* value <1e − 5, identity >70%).

### Phylogenetic tree construction and gene duplication or loss inference

The amino acid sequences of each Myb gene family were aligned using Mafft v7.471 with the maxiterate set as 1000^80^. FastTree (v2.1.11) software was used to perform phylogenetic analysis using the maximum-likelihood method^81^. The Jones-Taylor-Thorton model was adopted, and the bootstrap replications were set as 1000. The phylogenetic trees of each Myb gene family were illustrated using the iTOL program to add SSR-related information or gene function^67^. Gene duplication and gene loss analysis were performed using the Notung2.9 software^82^.

### Database construction

The PSSRD was constructed by applying various software packages, including MySQL database management, PHP, JavaScript, HTML, and CSS. The collected datasets were processed using Python or Perl, and several bioinformatics programs were used for interpreting biological data analysis and mining. The PSSRD contains several databases that store processed SSR-related data in MySQL. The interactive Web interface was constructed to enable users to conveniently access the PSSRD and obtain information for basic research using any popular browser on their devices. PHP, HTML, and JavaScript were used to transmit query requirements and extract data rapidly from the MySQL database to create report pages. The interactive plotting system was developed using d3.js and nvd3 helper libraries^83^. More importantly, two tools, WSF and BSF, are provided, which were rewritten according to the MISA^68^. These two tools will greatly facilitate the online or local batch identification of SSRs for users.

## Supplementary information

Supplementary Figures 1-7

Supplementary tables 1-12

## Data Availability

All related datasets in this study are available in our SSR database (PSSRD: http://www.pssrd.info/).

## Materials availability

All materials used in this study are available in our SSR database (PSSRD: http://www.pssrd.info/).
