# Supplementary material for: Comprehensive analysis of SSRs and database construction using all complete gene-coding sequences in major horticultural and representative plants
Source: Hortic Res. 2021 Jun 1;8:122. doi: 10.1038/s41438-021-00562-7 (PMC8167114; doi:10.1038/s41438-021-00562-7)
Supplement: Supplementary file 1 — Supplementary Figures 1-7 [file 41438_2021_562_MOESM1_ESM.docx]

**Supplementary Figures 1-7**

**Fig. S1 The workflow for comparative analysis of SSR and database construction in this study.**

**Fig. S2 Comparative analysis of different SSR characteristics among four groups of plants.** (a) The boxplot of SSR number. (b) The boxplot of the number of genes contained SSR. (c) The boxplot of percentage of genes contained SSR.

**
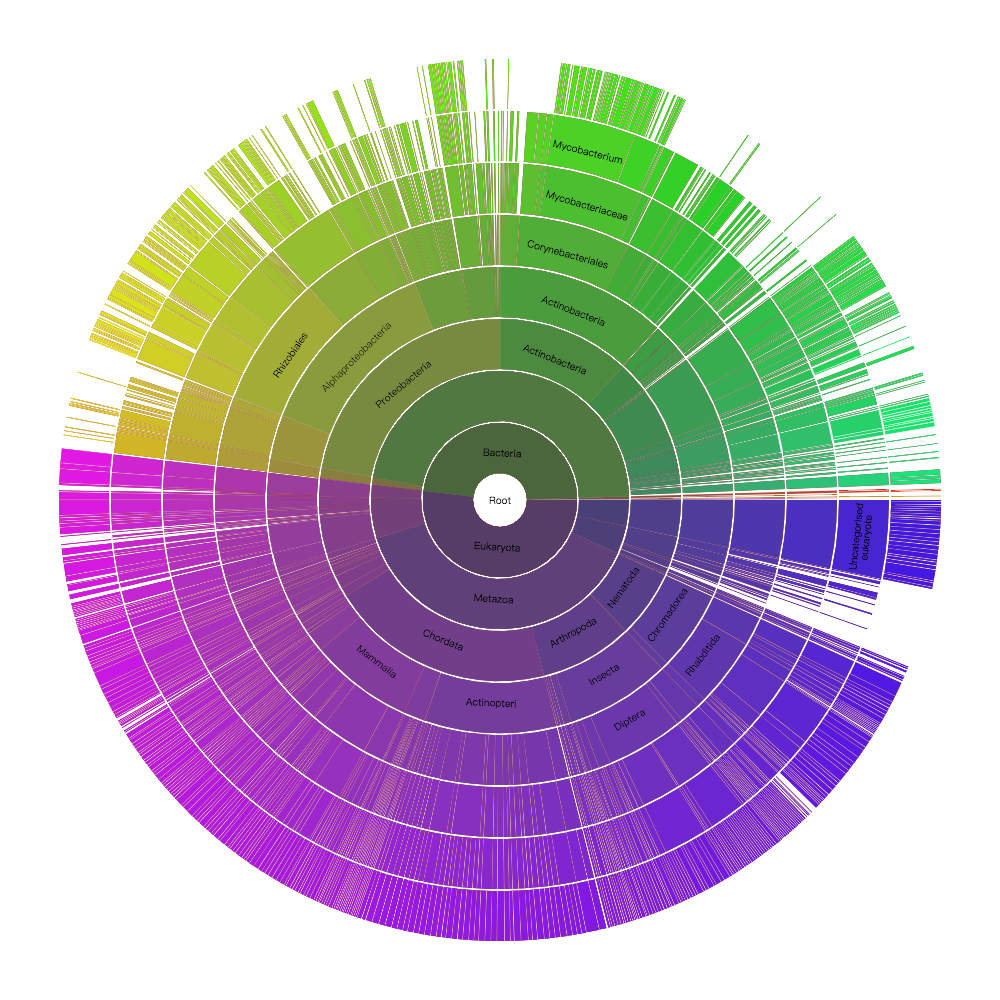
Fig. S3** The distribution of Guanylate_cyc (PF00211) domain in different kind of species according to the Pfam database.


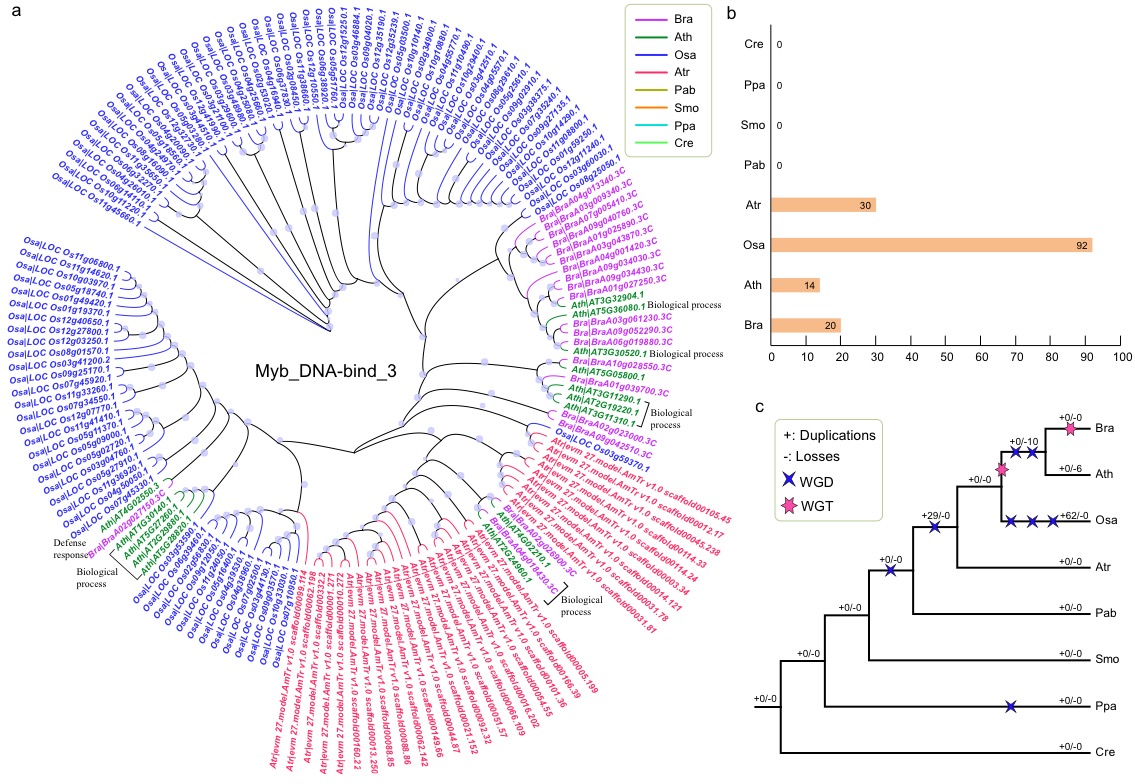


**Fig. S4 The phylogenetic and gene duplication or loss analysis of Myb_DNA-bind_3 gene family in 8 representative species.** (a) The maximum-likelihood (ML) trees were generated based on the amino acid sequences of Myb_DNA-bind_3 gene family. The phylogenetic tree was constructed using the FastTree software with 1000 bootstrap repeats in 8 species. The bootstrap values above 40% were shown with the circle on each branch. (b) The gene number of Myb_DNA-bind_3 gene family in each species. (c) The gene duplication and losses analyses of Myb_DNA-bind_3 gene family using Notung software in 8 species. Differential gene duplications and losses are indicated by numbers with + and - on each branch. WGD and WGT events are indicated with a quadrilateral and hexagon, respectively.


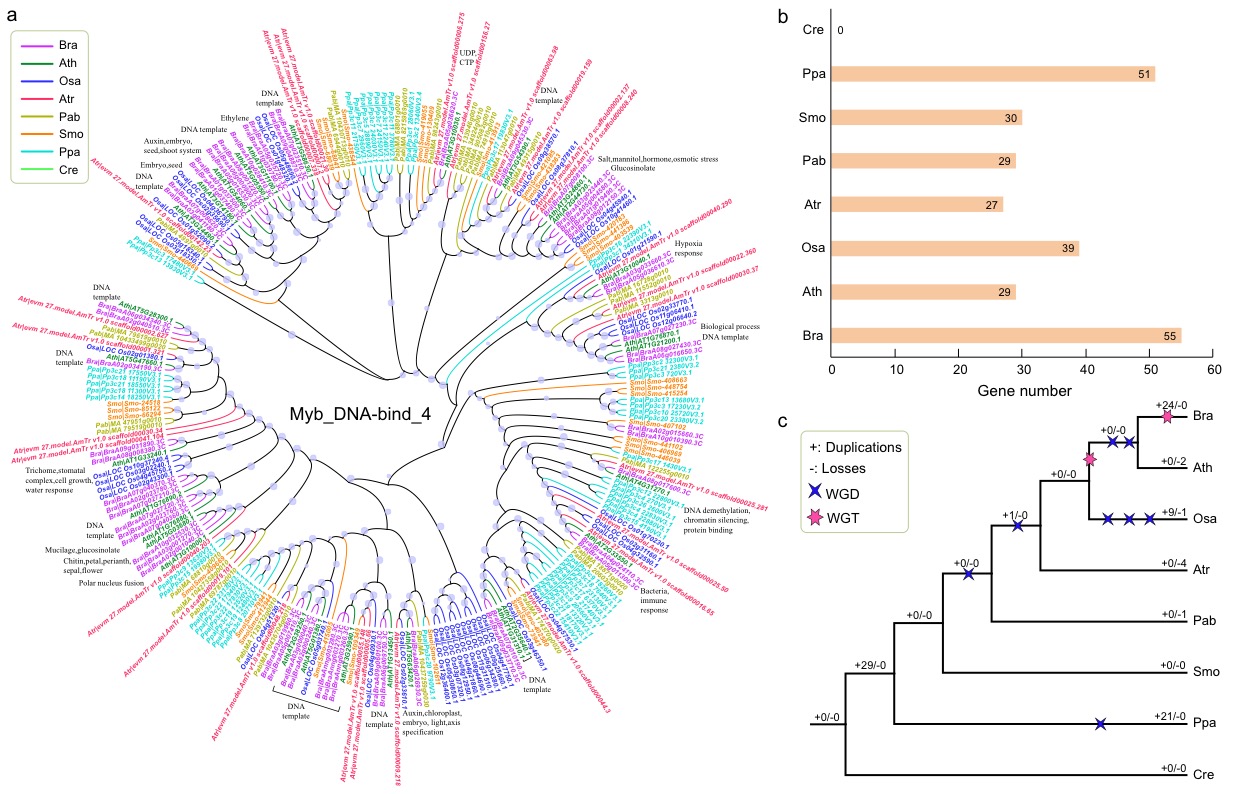
**Fig. S5 The phylogenetic and gene duplication or loss analysis of Myb_DNA-bind_4 gene family in 8 representative species.** (a) The maximum-likelihood (ML) trees were generated based on the amino acid sequences of Myb_DNA-bind_4 gene family. The phylogenetic tree was constructed using the FastTree software with 1000 bootstrap repeats in 8 species. The bootstrap values above 40% were shown with the circle on each branch. (b) The gene number of Myb_DNA-bind_4 gene family in each species. (c) The gene duplication and losses analyses of Myb_DNA-bind_4 gene family using Notung software in 8 species. Differential gene duplications and losses are indicated by numbers with + and - on each branch. WGD and WGT events are indicated with a quadrilateral and hexagon, respectively.

**
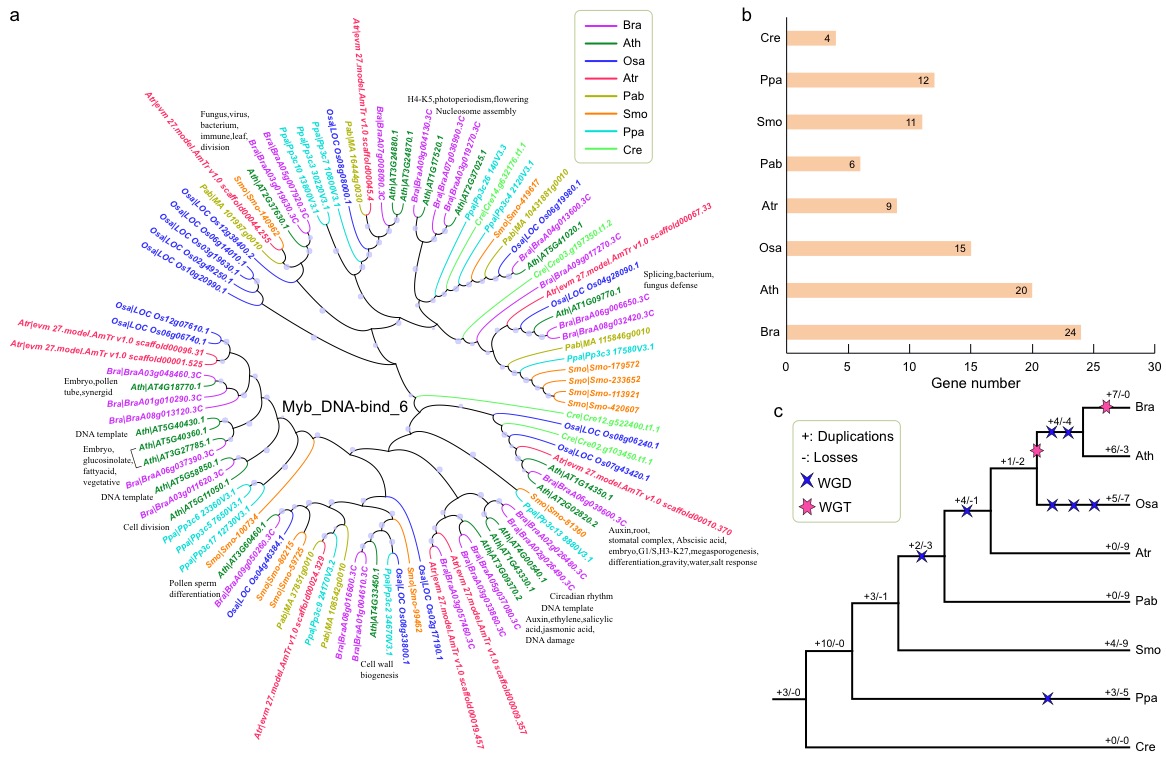
Fig. S6 The phylogenetic and gene duplication or loss analysis of Myb_DNA-bind_6 gene family in 8 representative species.** (a) The maximum-likelihood (ML) trees were generated based on the amino acid sequences of Myb_DNA-bind_6 gene family. The phylogenetic tree was constructed using the FastTree software with 1000 bootstrap repeats in 8 species. The bootstrap values above 40% were shown with the circle on each branch. (b) The gene number of Myb_DNA-bind_6 gene family in each species. (c) The gene duplication and losses analyses of Myb_DNA-bind_6 gene family using Notung software in 8 species. Differential gene duplications and losses are indicated by numbers with + and - on each branch. WGD and WGT events are indicated with a quadrilateral and hexagon, respectively.

**
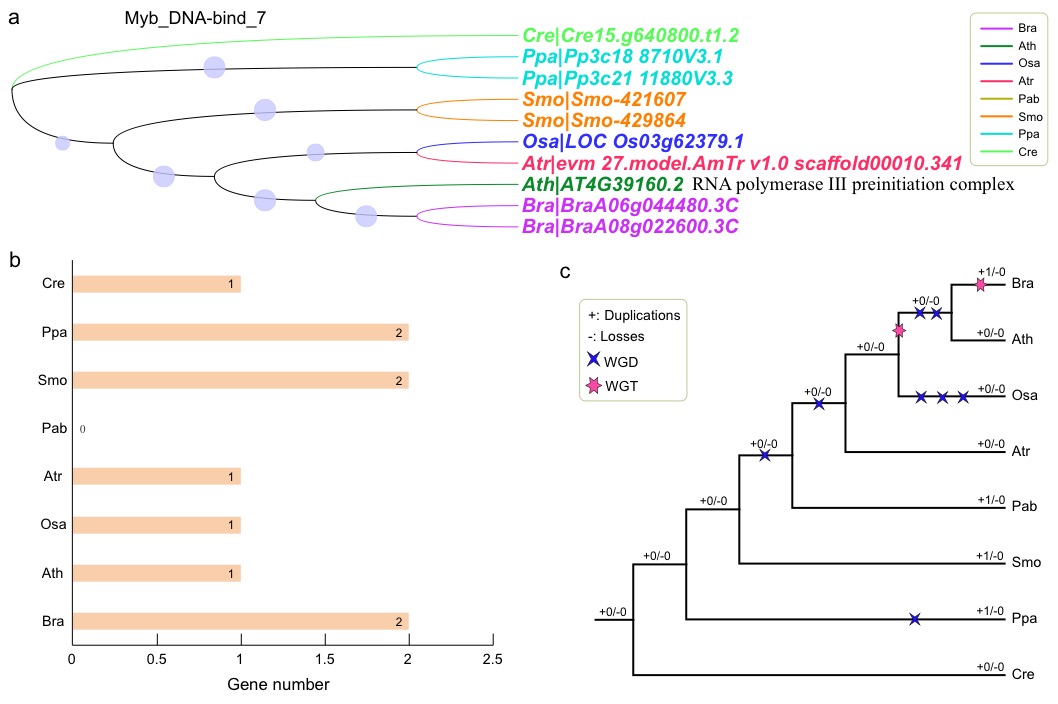
**

**Fig. S7 The phylogenetic and gene duplication or loss analysis of Myb_DNA-bind_7 gene family in 8 representative species.** (a) The maximum-likelihood (ML) trees were generated based on the amino acid sequences of Myb_DNA-bind_7 gene family. The phylogenetic tree was constructed using the FastTree software with 1000 bootstrap repeats in 8 species. The bootstrap values above 40% were shown with the circle on each branch. (b) The gene number of Myb_DNA-bind_7 gene family in each species. (c) The gene duplication and losses analyses of Myb_DNA-bind_7 gene family using Notung software in 8 species. Differential gene duplications and losses are indicated by numbers with + and - on each branch. WGD and WGT events are indicated with a quadrilateral and hexagon, respectively.
